# SIX4 Controls Anti-PD-1 Efficacy by Regulating STING Expression

**DOI:** 10.1158/2767-9764.CRC-23-0265

**Published:** 2023-11-27

**Authors:** Beiyuan Liang, Evan H. Zhang, Zhen Ye, Hayden Storts, Wei Jin, Xinru Zheng, Hannah Hylton, Olivia Zaleski, Xuanxuan Xing, Wayne Miles, Jing J. Wang

**Affiliations:** 1 Department of Cancer Biology and Genetics, The Ohio State University, Columbus, Ohio.; 2 Pelotonia Institute for Immuno-Oncology, James Comprehensive Cancer Center, Wexner Medical Center, The Ohio State University, Columbus, Ohio.

## Abstract

The cGAS/STING cytosolic DNA-sensing pathway plays a significant role in antitumor immunity. Expression of STING is tightly regulated and commonly reduced or defective in many types of cancer. We have identified SIX4 as a significant regulator of STING expression in colon cancer cells. We showed that knockout of SIX4 decreased STING expression at the mRNA and protein levels while ectopic expression of SIX4 increased STING expression. Depletion of SIX4 led to attenuated STING activation and downstream signaling. Reexpression of SIX4 or ectopic expression of STING in SIX4 knockout cells reversed the effect. Ectopic expression of SIX4 enhanced DMXAA and cGAMP-induced STING activation and downstream signaling. Importantly, decrease of SIX4 expression substantially decreased tumor infiltration of CD8^+^ T cells and reduced the efficacy of PD-1 antibodies to diminish tumor growth in immune competent mice *in vivo*. Finally, analysis of The Cancer Genome Atlas colon cancer dataset indicated that tumors with high SIX4 expression were significantly enriched in the Inflammatory Response pathway. SIX4 expression also correlated with expression of multiple IFN-stimulated genes, inflammatory cytokines, and CD8A. Taken together, our results implicate that SIX4 is a principal regulator of STING expression in colon cancer cells, providing an additional mechanism and genetic marker to predict effective immune checkpoint blockade therapy responses.

**Significance:**

Our studies demonstrate that SIX4 is an important regulator of STING expression, providing a genetic marker or a therapeutic target to predict or enhance immune checkpoint blockade therapy responses in colon cancer.

## Introduction

Cyclic GMP-AMP (cGAMP) synthase (cGAS) is a cytosolic DNA sensor that when activated generates cGAMP. cGAMP is a second messenger that triggers the stimulator of interferon genes (STING) adaptor to activate downstream signaling, including the Tank-binding kinase-1 (TBK1)/IRF3 and the IkB kinase of the NFκB signaling. Activation of IRF3 and NFκB induce the expression of type I IFNs and inflammatory cytokines and chemokines to initiate innate and adaptive immune responses (1). Numerous studies have demonstrated that DNA damage, genomic instability, and cellular stress activate cGAS/STING signaling (2, 3). Moreover, tumor cell–intrinsic cGAS/STING activation has been linked to efficient cancer treatment including radiation, chemotherapies, and anti-PD-1/PD-L1 therapies (4, 5). Potential mechanisms by which cGAS/STING activation enhances the efficacy of anti-PD-1/PD-L1 therapies include increasing T-cell infiltration in the tumors and/or reducing tumor-associated myeloid cells (5), elevating expression of MHC class I and tumor cell recognition by T cells (6), and enhancing the function of antigen-presenting cells, natural killer (NK) cells, and T cells (7–9). Interestingly, the cGAS/STING pathway is frequently suppressed in a variety of cancers (5, 10). Suppression of STING signaling represses immune response (11). The identification of strategies that restore or reactivate tumor cell–intrinsic cGAS/STING signaling may be useful to enhance cancer treatment efficacy.

Because it is an important regulator of innate immunity, STING expression is tightly controlled. Previous work has demonstrated that STING is regulated at the transcriptional, posttranscriptional, and posttranslational levels (6, 10, 12–15). For example, GATA1 and Sp3 have been shown to bind to the STING promoter and modify its transcription in NIH3T3 cells (12). Hypermethylation of the STING promoter is responsible for silenced STING expression in melanoma and colon cancers (6, 10). In addition, miRNA-576-3p has been identified as a negative regulator of STING expression that enables vesicular stomatitis virus (VSV) replication (13). Moreover, STING protein stability is regulated by ubiquitination, where USP18/USP20 increases STING stability by reducing its ubiquitination (14). STING activation has also been shown to be regulated by phosphorylation (15). Despite these studies, the regulation of STING expression *in vivo* remains largely unknown.

Sine oculis homeobox (SIX) 4 is a member of the SIX family of homeobox transcription factors (16). SIX4 expression is upregulated and has been shown to play a role in tumorigenesis and metastasis of lung, breast, colorectal cancer (17–19). Moreover, SIX4 regulates transcription of many target genes, such as YAP1 and c-MET in hepatocellular carcinoma as well as IDH1 in osteosarcoma (20, 21). SIX4 also appears to promote PI3K/AKT activation in colorectal cancer (18) and activates STAT3 in breast cancer (17).

Here we show that SIX4 is a key regulator of STING expression in colon cancer cells. Knockout of SIX4 significantly decreased STING expression at the mRNA and protein levels, whereas ectopic expression of SIX4 increased STING expression. Reducing SIX4 expression resulted in attenuated activation of STING signaling by STING agonists. Reexpression of SIX4 or ectopic expression of STING in SIX4 knockout cells reversed the effect. As expected, ectopic expression of SIX4 enhanced STING pathway activation. Importantly, depletion of SIX4 significantly reduced efficacy of tumor clearance mediated by an anti-PD-1 antibody in syngeneic immune competent mice *in vivo*. The reduction of SIX4-dependent tumor clearance was associated with decreased tumor infiltration of CD8^+^ T cells. Analysis of The Cancer Genome Atlas (TCGA) colon cancer dataset is consistent with a role for SIX4 in controlling STING-dependent tumor clearance. Taken together, our studies demonstrate that SIX4 controls STING expression and activation in colon cancer cells, providing an additional mechanism and a potential clinical genetic marker to predict effective response of patients with colon cancer to immune checkpoint blockade therapies.

## Materials and Methods

### Colon Cancer Cells

Mouse colon cancer cell lines, MC38 (RRID:CVCL_B288) and CT26 (RRID:CVCL_7256), and human colon carcinoma cell line, HT29 (RRID:CVCL_A8EZ), were purchased from ATCC. Human colon carcinoma cell line, TENN (RRID:CVCL_Y378), was established in Dr. Brattain's lab in 1981 (22). All cell lines were authenticated by short tandem repeat (STR) analyses at Ohio State University Genomics Shared Resources. STR profiles were cross-checked with the ATCC database. MC38, CT26, and HT29 cell lines displayed ≥ 80% match, which is considered valid (23). STR profile of TENN cell line has never been reported before. It was confirmed to be of human origin and contain no interspecies contamination. Cells were tested for *Mycoplasma* every 3 months with MycoAlert PLUS Mycoplasma Detection Kit (Lonza, catalog no. LT07-418).

Cells were maintained at 37°C in a humidified incubator with 5% CO_2_. MC38 and CT26 cells were cultured in DMEM supplemented with 10% FBS while HT29 and TENN cells in McCoy's 5A medium (Cytiva, catalog no. SH30200.01) with 10% FBS. Cells were passaged two to five times between thawing and use in the described experiments.

### Antibodies and Reagents

The information of antibodies used in this study is as following: Anti-SIX4 (Santa Cruz Biotechnology, catalog no. sc-390779, RRID: AB_3068563), anti-STING (Cell Signaling Technology, catalog no. 13647, RRID:AB_2732796), anti-phospho-STING (Cell Signaling Technology, catalog no. 72971, RRID:AB_2799831), anti-TBK1 (Cell Signaling Technology, catalog no. 3013, RRID:AB_2199749), anti-phospho-TBK1 (Cell Signaling Technology, catalog no.5483, RRID:AB_10693472), anti-ISG15 (Santa Cruz Biotechnology, catalog no. sc-166755, RRID:AB_2126308), anti-STAT1 (Cell Signaling Technology, catalog no. 9172, RRID:AB_2198300), anti-phospho-STAT1 (Cell Signaling Technology, catalog no. 9167, RRID:AB_561284), anti-Actin (Santa Cruz Biotechnology, catalog no. sc-47778 HRP, RRID:AB_2714189). The STING activator DMAXX and cGAMP were purchased from InvivoGen (catalog no. tlrl-dmx, tlrl-nacga23-1).

### Western Blot Analysis and Real-time Q-PCR

Whole-cell lysates were prepared in RIPA buffer (MilliporeSigma, catalog no. 20-188) supplemented with a protease inhibitor cocktail (Thermo Fisher Scientific, catalog no. 78430). Equivalent amounts of protein were separated by SDS-PAGE and transferred to a Nitrocellulose membrane (Bio-Rad, catalog no. 1620115). Proteins were detected using an enhanced chemiluminescence system (LI-COR Biosciences).

Total RNA was isolated, and reverse transcribed to cDNA. Q-PCR analysis was performed using PowerUp SYBR Green Master Mix (Thermo Fisher Scientific, catalog no. A25777). The primer sequences for mouse IFNβ, CXCL10, and STING are ATGAGTGGTGGTTGCAGGC-F, TGACCTTTCAAATGCAGTAGATTCA-R; AGTAACTGCCGAAGCAAGAA-F, GCACCTCCACATAGCTTACA-R and GGAACACCGGTCTAGGAAGC-F, TGGATCCTTTGCCACCCAAA-R, respectively. The primer sequences for human IFNβ, CXCL10 and STING are ATGACCAACAAGTGTCTCCTCC-F, GGAATCCAAGCAAGTTGTAGCTC-R; AGCAGAGGAACCTCCAGTCT-F, ATGCAGGTACAGCGTACAGT-R and CTTCACTTGGATGCTTGCC-F, CCCGTAGCAGGTTGTTGTAATG-R, respectively. Actin was used as an endogenous control.

### CRISPR/Cas9 Knockout of SIX4

The Alt-R CRISPR-Cas9 System (Integrated DNA Technologies) was used, in which guide RNA (gRNA) contains crRNA with specific DNA target sequence and tracrRNA labeled with ATTO 550 (ATTO-TEC). Two crRNAs targeting SIX4 (ACAACTCCACTCGGAACTTC and CCTCGCACACGCAGGCGACA) were synthesized. gRNAs complexed with CAS9 protein were transfected into MC38 cells by electroporation. ATTO 550-positive cells were sorted into pools by flow cytometry the next day. One week later, cells were harvested and SIX4 expression was measured by Western blot analysis to determine knockout efficiency.

### Plasmids, Transfection, and Infection

Mouse *Six4* cDNA is purchased from genomics online (catalog no. ABIN4003799) and cloned into pCDH-CMV-MCS-EF1α-Puro Cloning and Expression Lentivector from System Biosciences (catalog no. CD510B-1). Human *SIX4* cDNA was amplified from human cell genomic DNA by PCR and cloned into pCDH-CMV-MCS-EF1α-Puro vector. The expression vectors were cotransfeceted into 293T cells with psPAX2 (psPAX2 was a gift from Didier Trono, Addgene, catalog no. 12260, RRID:Addgene_12260) and pMD2.G (pMD2.G was a gift from Didier Trono, Addgene, catalog no. 12259, RRID:Addgene_12259). Supernatant containing viruses was collected and infected into colon cancer cells.

### STING Activator Treatment

MC38 and CT26 cells were treated with 10 µg/mL of DMXAA for 6 hours. Cells were harvest for protein and RNA isolation. HT29 and TENN cells were transfected with 10 µg/mL of cGAMP using lipofectamine 3000 (Thermo Fisher Scientific, catalog no. L300001). Six hours later, cells were harvested for protein and RNA isolation.

### Colony Formation Assays

Cells were seeded in 6-well plates at 400 cells per well. After 2 weeks, colonies were stained with MTT and dissolved in DMSO. Relative cell numbers were determined by spectrophotometric analysis at 570 nm.

### *In Vivo* Xenograft Model

Mouse experiments involving animals were approved by the Ohio State University Institutional Animal Care and Use Committee (IACUC) and IACUC regulations were followed. Exponentially growing MC38 cells (0.5 × 10^6^) were inoculated subcutaneously into the flank of 6-week-old C57BL/6J (RRID:IMSR_JAX:000664) mice on one side. Mice was randomly divided into two groups. On days 7, 10, and 13, each mouse was treated with 200 µg of either control IgG (ichorbio, catalog no. ICH2244, RRID:AB_2921379) or anti-PD-1 antibody (ichorbio, catalog no. ICH1091, RRID:AB_2921476) by intraperitoneal injection. Tumors were monitored and measured every other day. Tumor volumes (*V*) were calculated by the formula *V* = *W*^2^ × *L* × 0.5, where *W* represents the largest tumor diameter and *L* represents the next largest tumor diameter. Upon termination of the experiments, tumors were dissected out and photographed on the same scale.

### IHC Staining

Formalin-fixed paraffin-embedded blocks of tumors were cut into 4-µm-thick tissue sections. After antigen retrieval using Antigen Unmasking Solution (Vector Laboratories, catalog no. H-3300, RRID:AB_2336226), tissue slides were blocked with 10% Goat Serum (Vector Laboratories, catalog no. S-1000, RRID:AB_2336615) diluted in 1X PBS, followed by incubation with an anti-CD8 antibody (Cell Signaling Technology, catalog no. 98941, RRID:AB_2756376) overnight at 4°C. The slides were developed with ImmPACT DAB Substrate Kit (Vector Laboratories, catalog no. SK-4105, RRID:AB_2336520) and counterstained with hematoxylin (Leica biosystems, catalog no. 3801570). Five fields/slide were randomly selected for quantification by ImageJ (RRID:SCR_003070). Percentage of CD8^+^ cells was calculated as [(CD8^+^ cell number/total cell number in the field) × 100%].

### Bioinformatics Analysis of Clinical Data

RNA sequencing (RNA-seq) data for TCGA colon cancer dataset (TCGA-COAD) was accessed through the Genomic Data Commons Data Portal (GDC Data Portal; RRID:SCR_014514; https://portal.gdc.cancer.gov/; ref. 24). TCGA-COAD samples (*n* = 471) were classified in SIX4^high^ or SIX4^low^ based on SIX4 expression using z-score. Gene matrix files were modified to comply with .gct file format and used as input file for gene set enrichment analysis (GSEA, RRID:SCR_003199; refs. 25, 26). GSEA was performed with GSEA v4.3.2 module on GenePattern (ref. 27; https://cloud.genepattern.org/, Broad Institute) with 1,000 phenotype permutations using the MSigDb gene sets (28). Correlation of SIX4 with other genes was calculated with Spearman correlation. Analysis was conducted using R and RStudio.

### Statistical Analysis

Experiments were performed a minimum of two times independently and represented by mean ± SD. Statistical analysis was performed in GraphPad Prism 5 (RRID:SCR_002798). Student *t* test or ANOVA were used to analyze the differences among groups. Statistically significant differences are indicated as follows: *, *P* < 0.05; **, *P* < 0.01; ***, *P* < 0.001.

### Western Blot Quantification

LI-COR Image Studio Software (RRID:SCR_015795) was used for Western blot image acquisition and quantification. At least two sets of Western blots for each experiment were analyzed and the ratios of intensity values of specified proteins and respective actin were calculated. Mean ± SD were plotted. Statistics analysis was performed in GraphPad Prism 5 (RRID:SCR_002798) using Student *t* test.

### Data Availability Statement

The patient data analyzed in this study were obtained from https://portal.gdc.cancer.gov/. All other data in this article can be obtained from the corresponding author upon reasonable request.

## Results

### SIX4 Regulates STING Expression and Enhances the Activation of STING/TBK1/IFNβ Signaling

**FIGURE 1:**
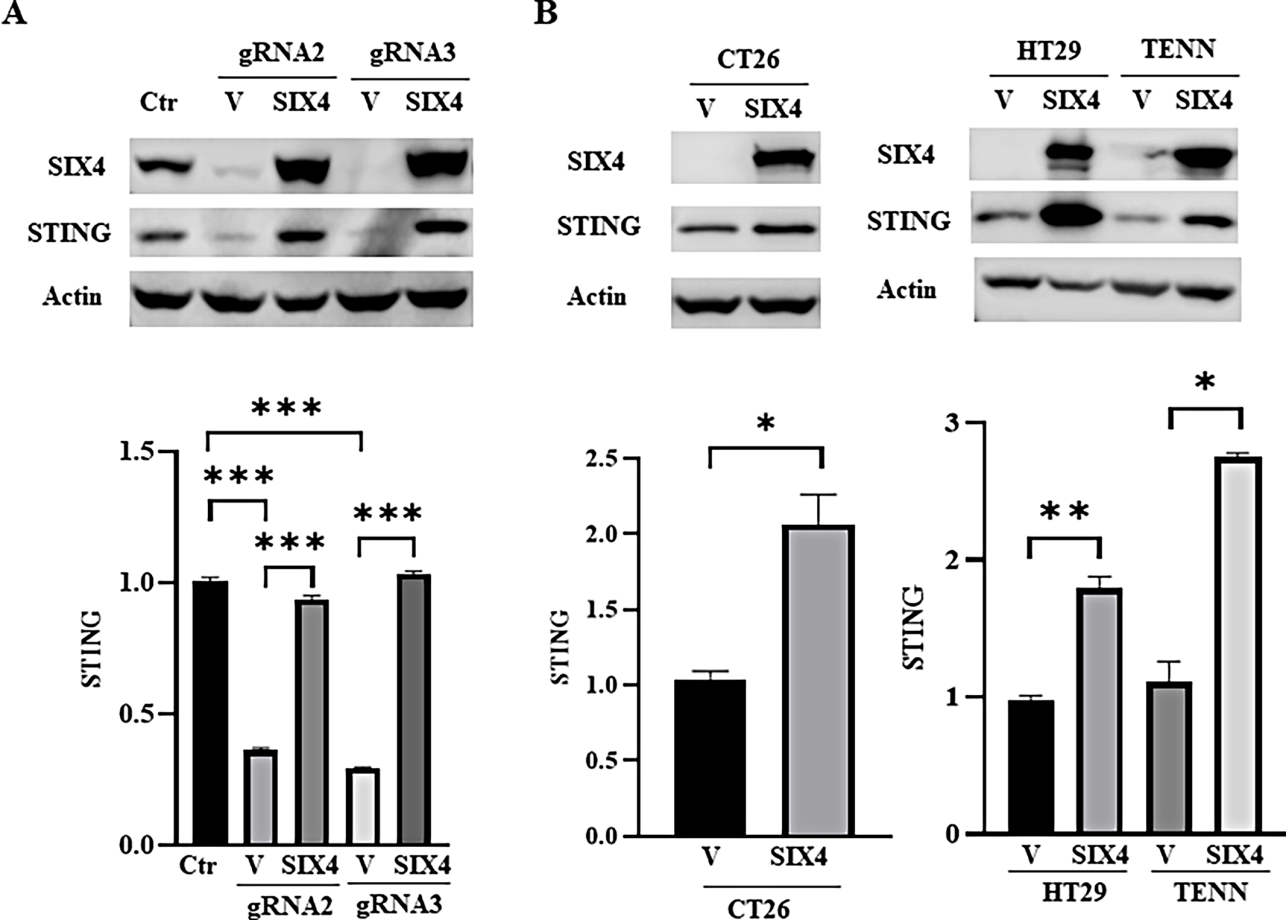
SIX4 regulates expression of STING mRNA and protein. **A,** SIX4 expression was knocked out in MC38 cells by two gRNAs (gRNA2 and gRNA3). An empty vector (V) or SIX4-expressing vector (SIX4) was introduced into SIX4 knockout cells. Depletion of SIX4 significantly reduced STING expression and reexpression of SIX4 rescued STING expression at mRNA and protein levels determined by Western blot (top) and Q-PCR (bottom) analysis, respectively. **B,** SIX4 was ectopically expressed in CT26, HT29, and TENN cells, which led to increased expression of STING mRNA and protein. Results are shown as mean ± SD. *, *P* < 0.05; **, *P* < 0.01; ***, *P* < 0.001.

To identify transcriptional regulators of STING, we used the JASPAR website (https://jaspar.genereg.net/) to identify potential STING regulators. We ascertained SIX4 as a promising candidate that might regulate STING expression. CRISPR/Cas9 and two distinct gRNAs were used to knock out SIX4 expression in MC38 cells. We found that deletion of SIX4 significantly reduced STING expression at both mRNA and protein levels (Fig. 1A; Supplementary Fig. S1A). Reexpression of SIX4 rescued STING expression (Fig. 1A; Supplementary Fig. S1A). Parallel studies showed that ectopic expression of SIX4 increased STING expression in CT26, HT29, and TENN cells (Fig. 1B; Supplementary Fig. S1B and S1C).

**FIGURE 2:**
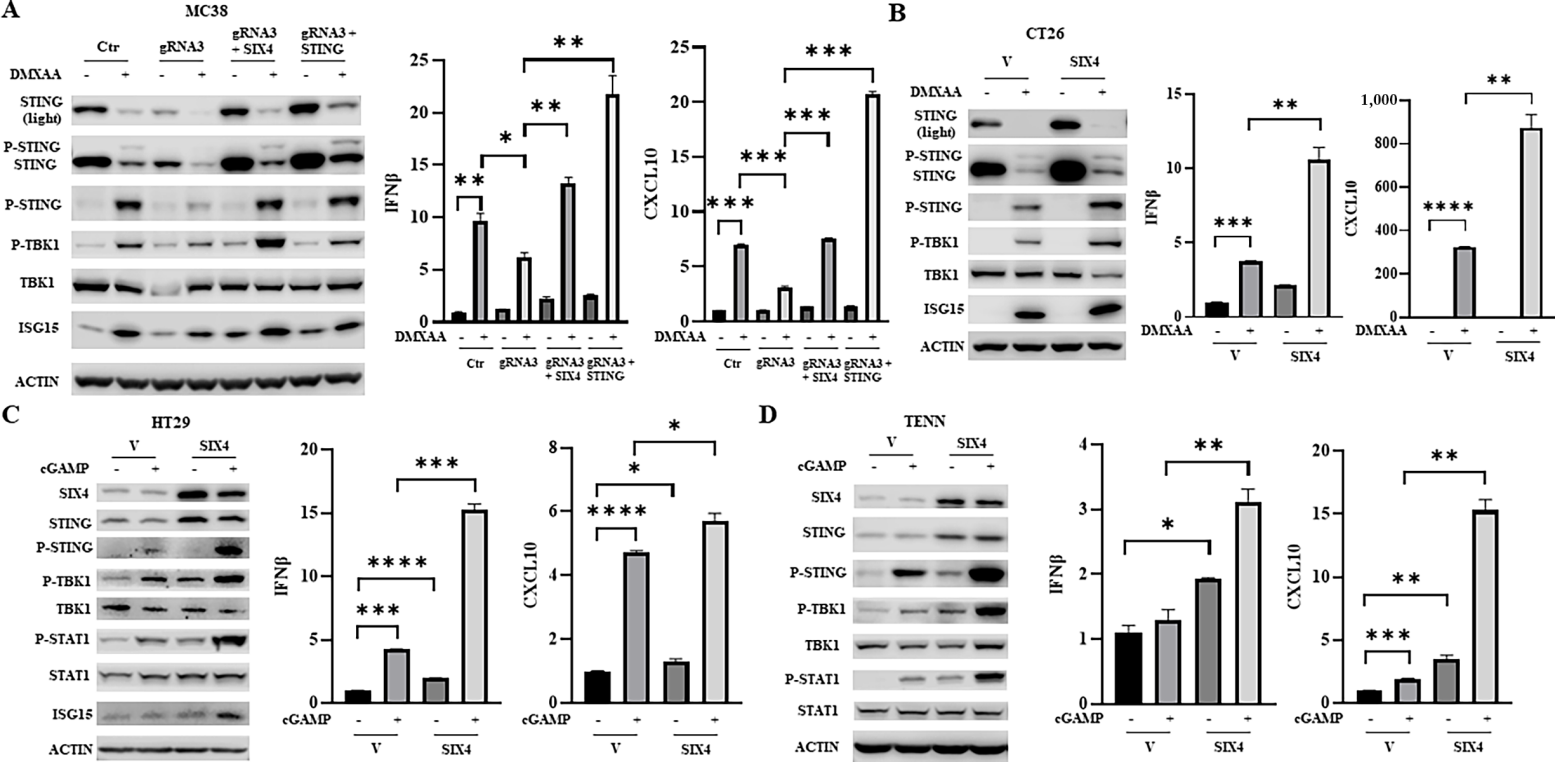
SIX4 modulates STING activation in colon cancer cells. **A,** MC38 SIX4 knockout cells and those with SIX4- or STING-reexpressing cells were treated with 10 µg/mL of DMXAA for 6 hours. Western blot analysis was performed to examine expression and phosphorylation of STING and TBK1 and ISG15 expression (left). IFNβ and CXCL10 mRNA expression was determined by Q-PCR analysis (right). **B,** CT26 control and SIX-overexpressing cells were treated with 10 µg/mL of DMXAA for 6 hours. Western blot analysis was performed to examine expression and phosphorylation of STING and TBK1 and ISG15 expression (left). IFNβ and CXCL10 mRNA expression was determined by Q-PCR analysis (right). The control and SIX4-overexpressing HT29 (**C**) and TENN (**D**) cells were transfected with 10 µg/mL of cGAMP. Cells were harvested for protein and RNA isolation. Western blot analysis was performed to examine expression and phosphorylation of STING, TBK1 and STAT1 and ISG15 expression (left). IFNβ and CXCL10 mRNA expression was determined by Q-PCR analysis (right). Results are shown as mean ± SD. *, *P* < 0.05; **, *P* < 0.01; ***, *P* < 0.001; ****, *P* < 0.0001.

Activation of STING and downstream signaling results in the production of type I IFNs and inflammatory cytokines (1). Treatment of MC38 cells with DMXAA, a specific STING agonist, resulted in significant upregulation of STING and TBK1 phosphorylation as well as elevated expression of IFNβ, CXCL10, and IFN-stimulated gene 15 (ISG15), an IFN-regulated gene (Fig. 2A; Supplementary Fig. S2A). Depletion of SIX4 attenuated DMXAA-mediated STING activation, while reexpression of either SIX4 or STING restored the effect of DMXAA effect (Fig. 2A; Supplementary Fig. S2A). Similarly, ectopic expression of SIX4 in CT26 cells further enhanced DMXAA-mediated activation of STING signaling and increased expression of IFNβ, CXCL10, and ISG15 (Fig. 2B; Supplementary Fig. S2B). We noted that STING protein levels were significantly reduced by DMXAA treatment (Fig. 2A and B). This reduction was accompanied by the appearance of a higher molecular weight band, which is phosphorylated STING (P-STING; Fig. 2A and B; refs. 15, 29). Previously studies have demonstrated that STING activation leads to STING phosphorylation and subsequently to the degradation of STING (15, 29). Our observations are consistent with these studies and suggest that acute activation of STING leads to reduction of STING expression as a negative feedback control of STING activity to counteract sustained innate immune signaling (15, 29).

To determine whether SIX4 regulates STING/TBK1/IFNβ signaling in human colon cancer cells, HT29 and TENN cells were transfected with cGAMP to activate STING in the presence or absence of SIX4 overexpression. Increased expression of SIX4 enhanced well-known cGAMP-mediated increases in STING, TBK1, and STAT1 phosphorylation as well as upregulation of expression of IFNβ, CXCL10, and ISG15 (Fig. 2C and D; Supplementary Fig. S3 and S4). Taken together, these results indicate that SIX4 activates STING/TBK1/IFNβ signaling through upregulation of STING expression.

### Reduction of SIX4 Expression Reduces Anti–PD-1–Mediated Suppression of Tumor Growth *In Vivo*

**FIGURE 3:**
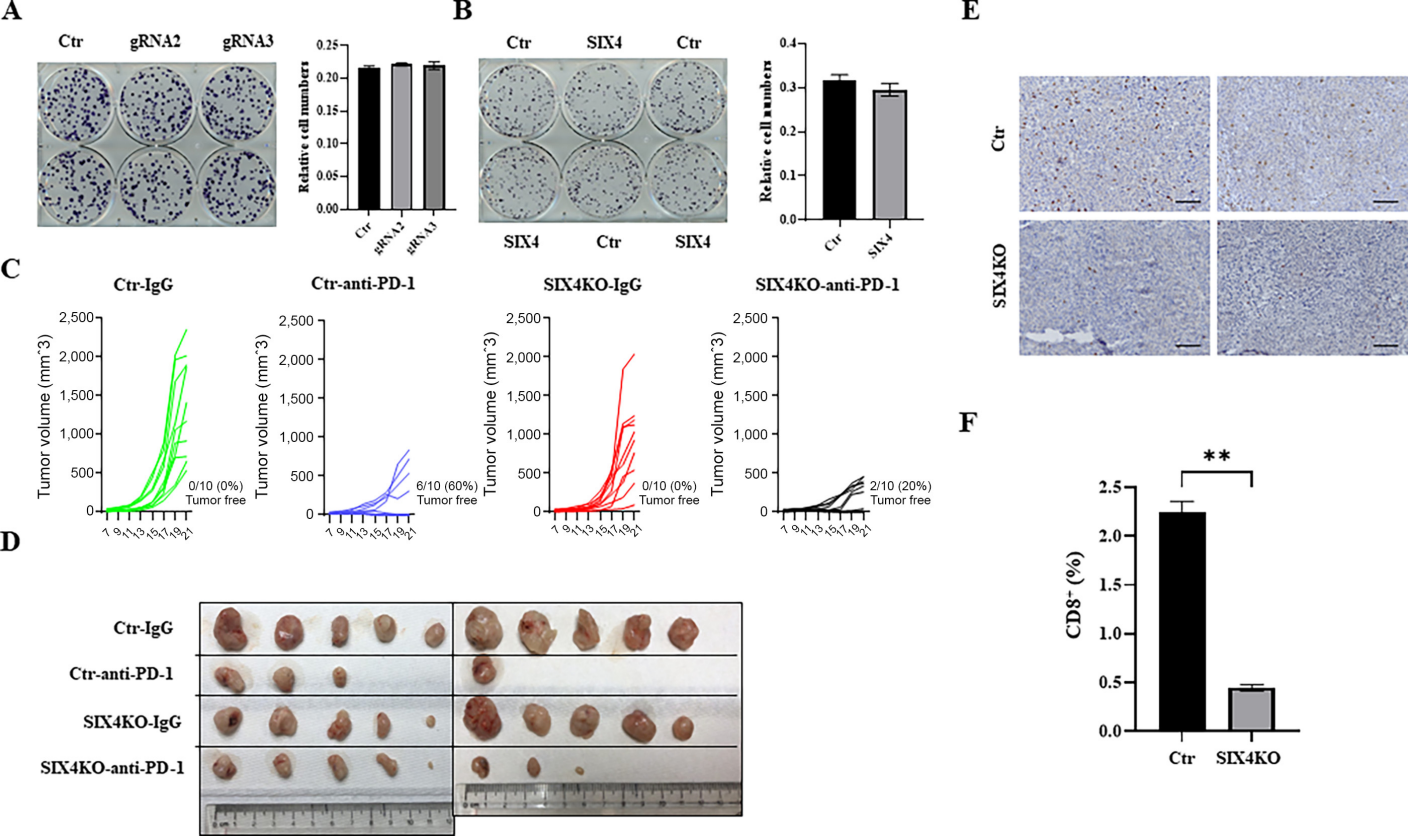
Knockout of SIX4 reduces the efficacy of anti-PD-1 tumor suppression effect *in vivo*. **A,** Colony formation assays were performed in MC38 control and SIX4 knockout cells (gRNA2 and gRNA3) with representative images after MTT staining (left). After dissolving in DMSO, relative cell numbers were determined by spectrophotometry at 570 nm (right). **B,** Colony formation assays were performed in MC38 control and SIX4-overexpressing cells with representative images after MTT staining (left). After dissolving in DMSO, relative cell numbers were determined by spectrophotometry at 570 nm (right). **C,** Xenograft tumor growth curves of MC38 control and SIX4 knockout (KO) cells treated with a control IgG or an anti-PD-1 antibody are shown. *N* = 10. **D,** Images of tumors at the endpoint of experiments (day 21) are shown. The unit of the rulers is cm. **E,** Representative images of IHC staining of CD8 in control and SIX4 knockout tumors. Scale bars: 100 µm. **F,** Quantification of percentage of CD8-positive cells. Results are shown as mean ± SD. **, *P* < 0.01.

Previous work has suggested SIX4 enhances cell proliferation in tumors (16). To establish whether SIX4 expression affects cell proliferation, we performed colony formation analysis in control, SIX4 knockout and SIX4-overexpressing MC38 cells. The results revealed that depletion or overexpression of SIX4 had trivial effect on the clonogenic growth of MC38 cells in culture (Fig. 3A and B).

Given the importance of STING activation in the efficacy of anti-PD-1/PD-L1 therapies (4), we next determined the effect of SIX4 expression on the efficiency of an anti-PD-1 antibody-mediated inhibition of tumor growth in immune competent mice. Control and SIX4 knockout MC38 cells were injected subcutaneously into wild type C57BL/6 mice that were then randomly divided into two groups and treated with a normal IgG or an anti-PD-1 antibody, respectively. Although depletion of SIX4 did not have a significant effect on tumor growth, it substantially reduced the response to anti-PD-1 treatment. Treatment with normal IgG in both control and SIX4 knockout tumors resulted in 100% tumor growth. Notably, treatment with the anti-PD-1 antibody led to complete disappearance of tumors in 6 out 10 mice bearing control cells whereas it only cleared tumors in 2 out of 10 mice bearing SIX4 knockout cells (Fig. 3C and D). It has been shown previously that activating STING by STING-specific agonists improves anti–PD-1–mediated suppression effect in MC38 tumor model (30). Our results suggest that, by regulating STING expression, SIX4 contributes to the efficacy of anti-PD-1 therapy.

One of the mechanisms by which tumor cell–intrinsic STING activation enhances anti-PD-1/PD-L1 therapies is by increasing intratumoral T cells (5). IHC staining of CD8 in tumor sections showed a marked reduction of CD8-positive T cells in the tumors of SIX4 knockout cells as compared with control tumors (Fig. 3E). Quantification indicated that the percentage of CD8^+^ T cells in SIX4 knockout tumors was reduce to 22% of that in control tumors (Fig. 3F). Taken together, these results indicate that depletion of SIX4 reduced CD8^+^ T-cell infiltration and attenuated tumor response to anti-PD-1 treatment.

### SIX4 Expression is Associated with Inflammatory Response in Colon Cancer Patient Specimens

**FIGURE 4:**
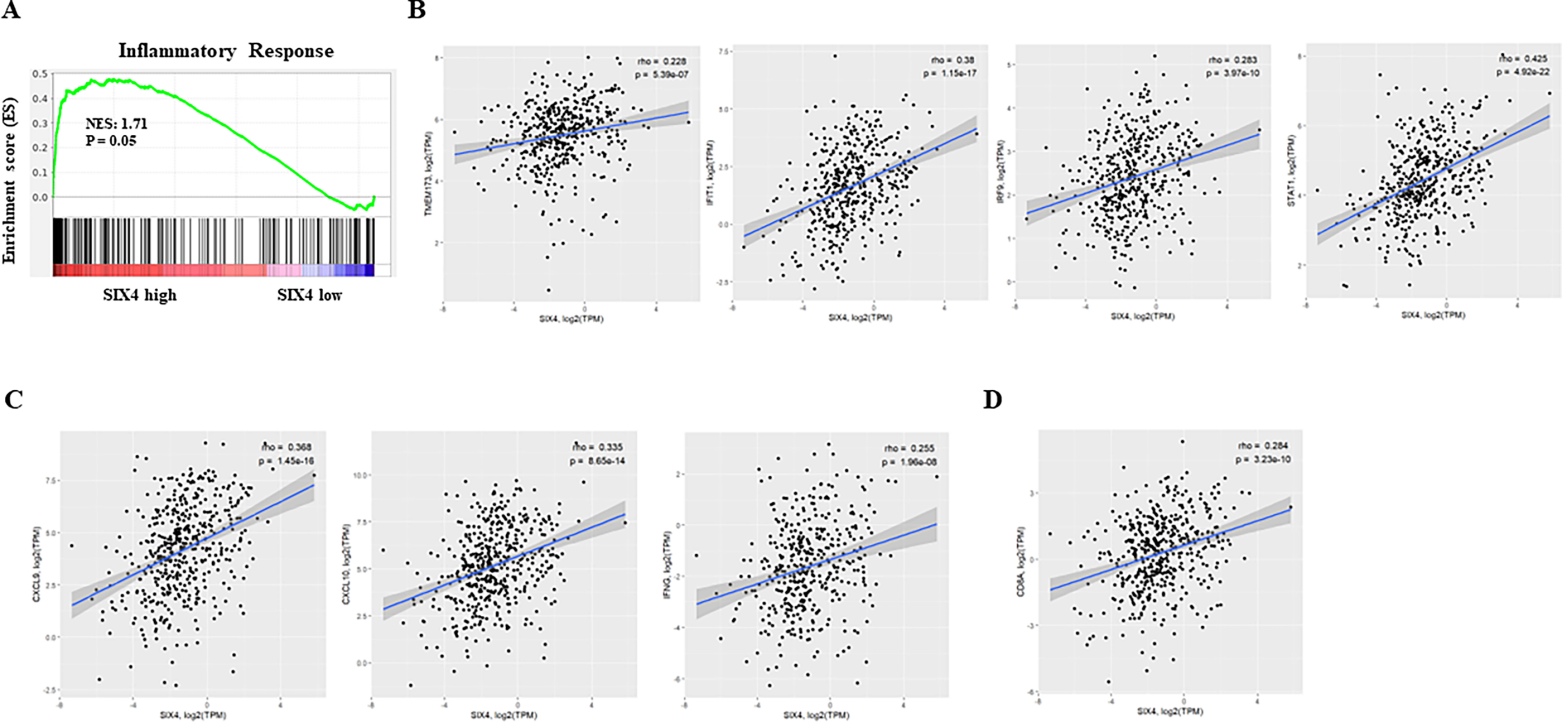
SIX4 expression is positively associated with inflammatory response in colon cancer patient specimens. **A,** GSEA plots of enrichment in the Inflammatory Response signatures from MsigDB in colon cancer patient samples from TCGA stratified by SIX4 expression. **B,** Correlation between SIX4 and STING or IFN marker gene expression in colon cancer patient samples from TCGA. **C,** Correlation between SIX4 and inflammatory cytokine/chemokine expression in colon cancer patient samples from TCGA. **D,** Correlation between SIX4 and CD8A expression in colon cancer patient samples from TCGA.

We examined the relationship between SIX4 expression and STING-dependent inflammatory activities in human colon cancer by mining RNA-seq data from TCGA-COAD. Patients were stratified into SIX4 high and low expression groups. Differential gene expression was determined between two groups and GSEA was performed. The SIX4 high expression group was significantly enriched in the Inflammatory Response pathway (Fig. 4A). In addition, SIX4 expression was significantly correlated with expression of STING, multiple ISGs (Fig. 4B), inflammatory cytokines (Fig. 4C) and CD8A that is predominantly expressed on cytotoxic T cells (Fig. 4D). These results suggest that high SIX4 expression in patients with colon cancer likely results in elevated STING expression, enhanced STING activation, inflammatory response, and increased infiltration of CD8^+^ lymphocytes compared with low SIX4 expression. These results appear largely identical to our *in vitro* and *in vivo* results using colon cancer cell lines presented here.

## Discussion

Previous studies have suggested that SIX4 function is correlated with tumor-promoting activity (17–21). Our studies demonstrate that SIX4 enhances STING/IFNβ innate immune signaling by upregulating STING expression. Tumor cell–intrinsic STING activation plays an important role in antitumor immunity by increasing tumor-infiltrating T cells, reducing tumor-associated myeloid cells and/or enhancing tumor antigenicity and T-cell recognition (5, 6). This tumor suppression activity of SIX4 adds an additional dimension to the SIX4 function during tumorigenesis.

SIX4 expression has been shown to be upregulated in colorectal cancer (18). High SIX4 expression is likely to increase STING expression based on our studies. Nevertheless, it appears that the STING promoter is hypermethylated in some colorectal tumors, leading to silencing of STING expression (10). In STING-silenced tumors, SIX4 would be unable to upregulate STING expression. In that case treatment with demethylating agents such as azacytidine or decitabine may restore upregulation of STING expression by SIX4. Taken together, these observations strongly suggest that tumor cells may evolve multiple mechanisms to escape SIX4’s upregulation of the STING pathway and tumor suppression function ultimately facilitating its tumor-promoting activity. Our analysis of colon cancer patient data indicated that tumors with high SIX4 expression were significantly enriched in the Inflammatory Response pathway and that SIX4 expression was positively associated with CD8A expression. It seems likely that patients with colon cancer with high SIX4 expression may have immunogenic tumors with infiltration of CD8^+^ T cells. These patients are likely to respond well to the anti-PD-1/PD-L1 treatment. Furthermore, for those patients with methylated STING promoter, the combination of anti-PD-1/PD-L1 with demethylating agents (e.g., azacytidine or decitabine) would likely improve the clinical treatment efficacy.

Our studies showed that SIX4 knockout tumors grew similarly as the control tumors with control IgG treatment, indicating that SIX4 expression by itself has insignificant effect on tumor growth in syngeneic mouse model. However, anti-PD-1 treatment showed lower tumor clearance rate in SIX4 knockout group than in control group even though the treatment reduced tumor growth in both control and SIX4 knockout groups with tumors remaining. These observations suggest that the PD-1 antibody operates more efficiently in control tumors to eliminate cancer cells at the beginning of the treatment than in SIX4 knockout tumors and that knockout of SIX4 may not be able to completely deplete STING expression. Moreover, cancer cells that survived the anti-PD-1 treatment probably developed escaping mechanism(s) and continued to grow regardless of SIX4 expression. Identifying additional mechanisms by which cancer cells evade immune regulation is an important and active field of research. Ultimately, these studies may shed light on the mechanisms of patients’ resistance to immune checkpoint blockade therapies and facilitate development of strategies to overcome the resistance.

In summary, we have identified SIX4 as a novel regulator of STING expression, which displays a tumor suppression function by increasing antitumor immunity. Knockout of SIX4 attenuated STING activator–mediated activation of STING/IFNβ signaling cascade and reduced efficacy of an anti-PD-1 antibody to eliminate tumor growth in immune competent mice. Data analysis of human patients with colon cancer support the hypothesis that SIX4 increases STING/type I IFN signaling and enhances inflammatory responses, which facilitates T-cell infiltration and antitumor immunity. SIX4 could regulate STING expression directly by binding to its promoter or indirectly through its target genes. Additional studies will be required to distinguish these possibilities. Nevertheless, SIX4 regulation of STING expression appears to play a significant role in colon cancer. Therefore, our studies add an additional layer to SIX4 function in regulating colon cancer progression and identify it as a potential target for enhancing immunotherapy.

## Supplementary Material

Supplemental Figure 1 Supplemental Figure 1 shows the quantification of western blots shown in Figure 1A and 1B.

Supplemental Figure 2 Supplemental Figure 2 shows the quantification of western blots shown in Figure 2A and 2B.

Supplemental Figure 3 Supplemental Figure 3 shows the quantification of western blots shown in Figure 2C.

Supplemental Figure 4 Supplemental Figure 4 shows the quantification of western blots shown in Figure 2D.
