# Supplementary material for: SIX4 Controls Anti-PD-1 Efficacy by Regulating STING Expression
Source: Cancer Res Commun. 2023 Nov 27;3(11):2412–9. doi: 10.1158/2767-9764.CRC-23-0265 (PMC10680432; doi:10.1158/2767-9764.CRC-23-0265)
Supplement: Supplemental Figure 1 — shows the quantification of western blots shown in Figure 1A and 1B. [file crc-23-0265-s01.pdf]

Supplemental Fig. 1

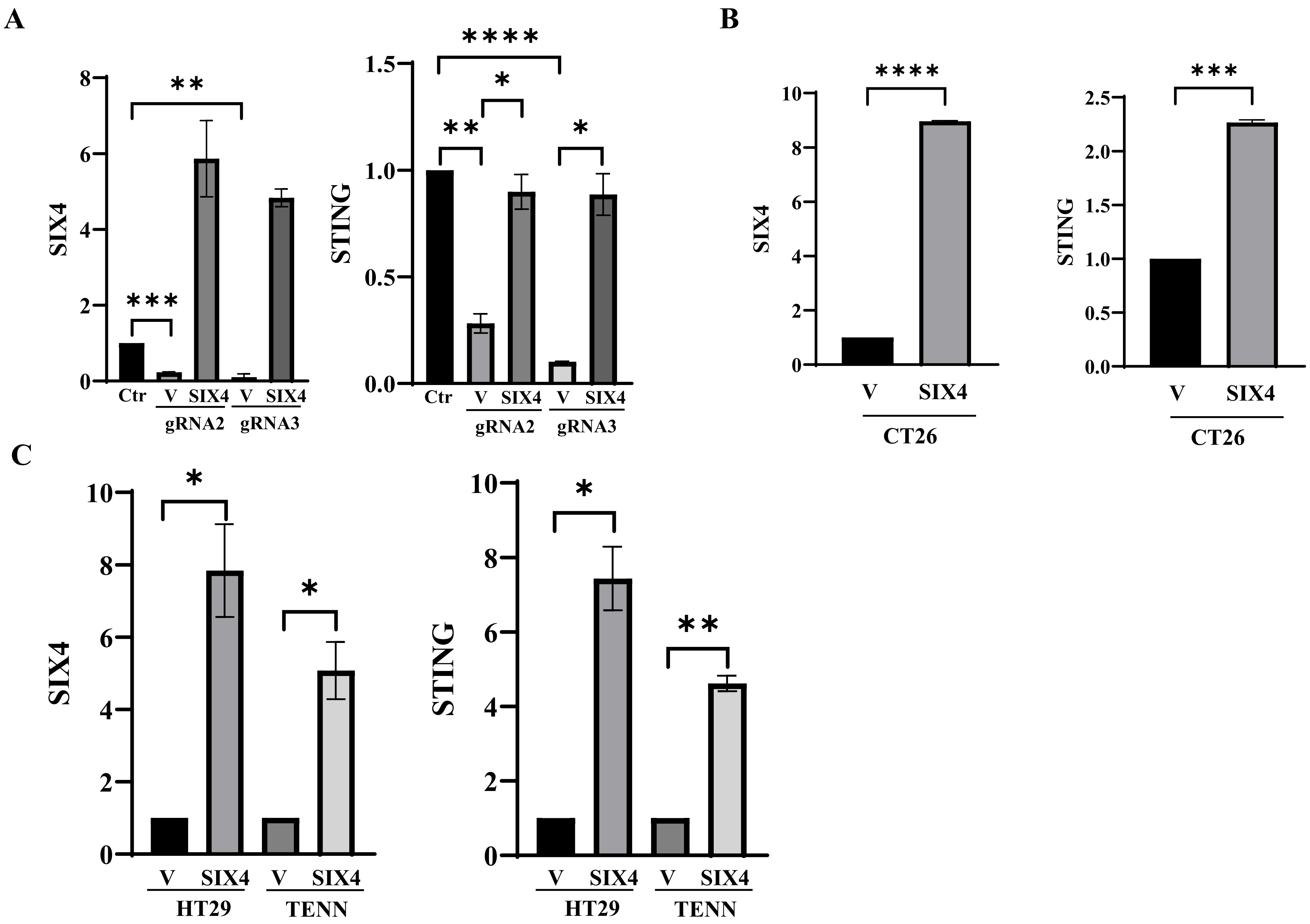

Supplemental Figure 1. **Quantification of western blots shown in Figure 1A and 1B.** A, Quantification of western blots of SIX4 and STING in MC38 cells as shown in Fig. 1A. B, Quantification of western blots of SIX4 and STING in CT26 cells as shown in Fig. 1B. C, Quantification of western blots of SIX4 and STING in HT29 and TENN cells as shown in Fig. 1B.
