# Supplementary material for: SIX4 Controls Anti-PD-1 Efficacy by Regulating STING Expression
Source: Cancer Res Commun. 2023 Nov 27;3(11):2412–9. doi: 10.1158/2767-9764.CRC-23-0265 (PMC10680432; doi:10.1158/2767-9764.CRC-23-0265)
Supplement: Supplemental Figure 2 — shows the quantification of western blots shown in Figure 2A and 2B. [file crc-23-0265-s02.pdf]

Supplemental Fig. 2

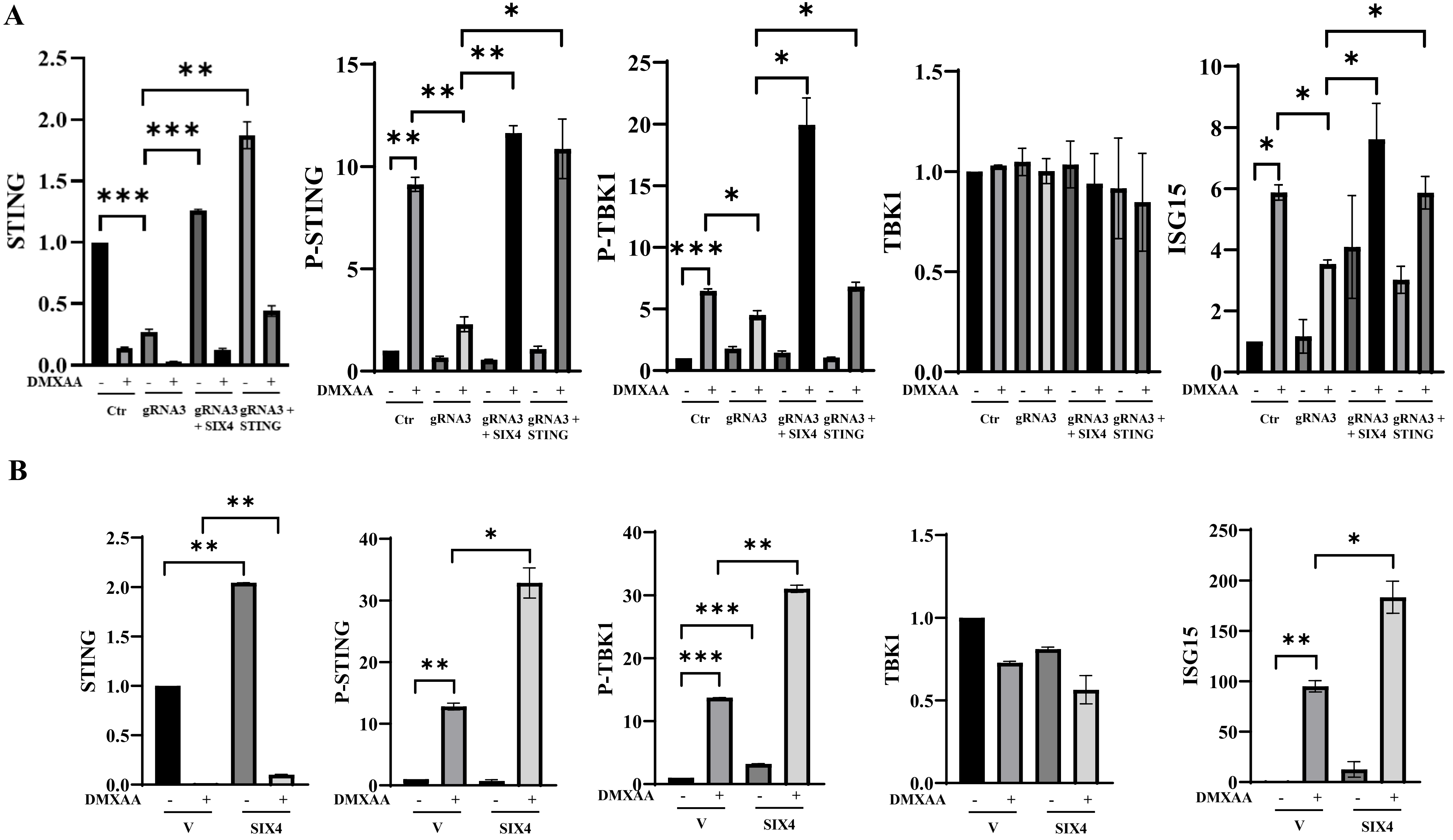

Supplemental Figure 2. **Quantification of western blots shown in Figure 2A and 2B.** A, Quantification of western blots of STING, P-STING, P-TBK1, TBK1 and ISG15 in MC38 cells as shown in Fig. 2A. B, Quantification of western blots of STING, P-STING, P-TBK1, TBK1 and ISG15 in CT26 cells as shown in Fig. 2B.
