# Supplementary material for: SIX4 Controls Anti-PD-1 Efficacy by Regulating STING Expression
Source: Cancer Res Commun. 2023 Nov 27;3(11):2412–9. doi: 10.1158/2767-9764.CRC-23-0265 (PMC10680432; doi:10.1158/2767-9764.CRC-23-0265)
Supplement: Supplemental Figure 3 — shows the quantification of western blots shown in Figure 2C. [file crc-23-0265-s03.pdf]

Supplemental Fig. 3

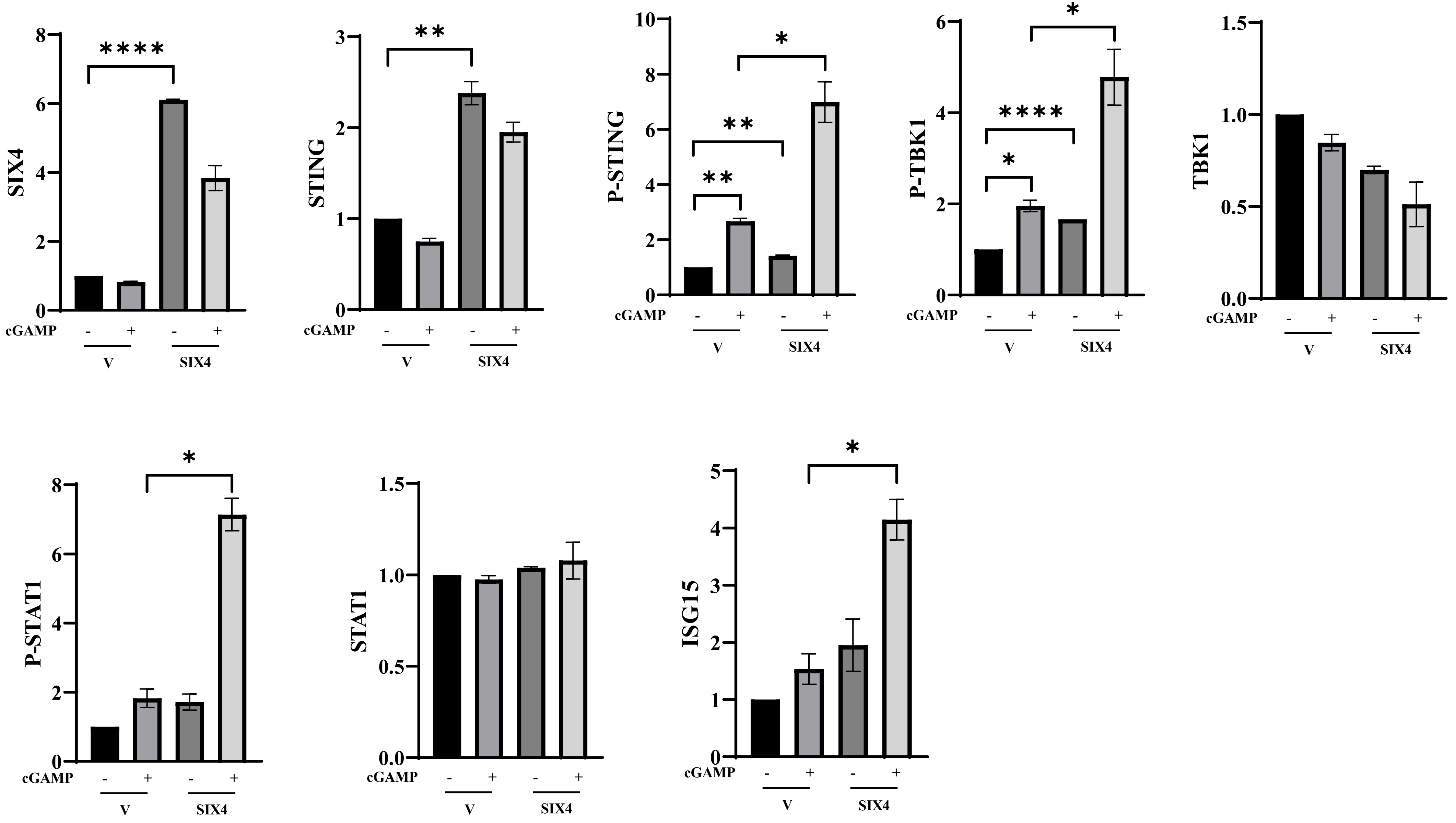

Supplemental Figure 3. **Quantification of western blots shown in Figure 2C.** Quantification of western blots of SIX4, STING, P-STING, P-TBK1, TBK1, P-STAT1, STAT1 and ISG15 in HT29 cells as shown in Fig. 2C.
